# Predictive risk factors for postoperative pneumonia after heart transplantation

**DOI:** 10.1186/s12871-019-0923-3

**Published:** 2020-01-07

**Authors:** Charles Vidal, Romain Pasqualotto, Arthur James, Pauline Dureau, Julie Rasata, Guillaume Coutance, Shaida Varnous, Pascal Leprince, Julien Amour, Adrien Bouglé

**Affiliations:** 1 Department of Anesthesiology and Critical Care Medicine, Pitié-Salpêtrière Hospital, Sorbonne Université, UMR INSERM 1166, IHU ICAN, Paris, France; 2 Department of Cardiovascular and Thoracic Surgery, Pitié-Salpêtrière Hospital, Sorbonne Université, UMR INSERM 1166, IHU ICAN, Paris, France; 3 Réanimation polyvalente, Centre Hospitalier Universitaire Félix Guyon, Allée des Topazes, 97400 Saint Denis de la Réunion, France

**Keywords:** Pneumonia, Heart transplantation, Sensitized recipient, Transfusion, Mechanical ventilation

## Abstract

**Background:**

Pneumonia is a frequent complication in patients undergoing heart transplantation (HTx) that increases morbidity and mortality in this population. Nevertheless, the risk factors for postoperative pneumonia (POP) are still unknown. The aim of this study was to investigate the predictive risk factors for POP in HTx recipients.

**Methods:**

In this retrospective study, all patients undergoing HTx between January 2014 and December 2015 were included. All cases of POP occurring until hospital discharge were investigated. The study aimed to determine risk factors using univariate and multivariate Cox regression models. Data are expressed in Odds Ratio [95% CI]. *P* < 0.05 was necessary to reject the null hypothesis.

**Results:**

A total of 175 patients were included without any patients being lost to follow-up, and 89 instances of POP were diagnosed in 59 (34%) patients. *Enterobacteriaceae* and *Pseudomonas aeruginosa* were the most common pathogens. In the multivariate analysis, the risk factors were preoperative mechanical ventilation (OR 1.42 [1.12–1.80], *P* < 0.01) and perioperative blood transfusion (OR 1.42 [95% CI: 1.20–1.70], *P* < 0.01). POP significantly impacted mortality at 30 days (OR: 4 [1.3–12.4], *P* = 0.01) and 1 year (OR: 6.8 [2.5–8.4], *P* < 0.01) and was associated with a longer duration of mechanical ventilation, time to weaning from venoarterial extracorporeal membrane oxygenation and stay in an intensive care unit. Plasma exchanges and intravenous administration of immunoglobulins did not increase the risk of POP.

**Conclusion:**

After HTx, preoperative mechanical ventilation and blood transfusion were risk factors for POP and were associated with increased mortality. *Enterobacteriaceae* and *Pseudomonas aeruginosa* are the most common pathogens of POP.

## Background

Heart transplantation (HTx) is still the gold standard for the treatment of chronic heart failure, improving not only the survival but also the quality of life of these patients [1]. The administration of an immunosuppressive treatment is essential to prevent rejection, but these therapies expose the HTx recipient to infectious bacterial, viral and fungal complications [2]. Thus, during the first year following HTx, postoperative infections represent the main cause of death in HTx recipients [3]. In cardiac surgery, POP is still the most common infectious complication [4] and is known to be associated with increased mortality [5, 6]. After HTx, the incidence and risk factors for POP remain poorly investigated [7–10]. In addition, plasmapheresis and intravenous immunoglobulins (IVIg) used to prevent antibody-mediated rejection in sensitized recipients may also influence the occurrence of POP after HTx [11–13]. The aim of this study was to investigate the risk factors for POP in HTx recipients, with or without preformed donor-specific anti-HLA antibodies (pfDSA).

## Methods

### Inclusion criteria

This is a retrospective study conducted in the academic centre of Pitié-Salpêtrière in Paris, France. All patients undergoing HTx, with or without pfDSA, between January 1st, 2014, and December 31th, 2015, were included in the study. This work complies with the Declaration of Helsinki, and the institutional review board approved the protocol (IRB 00010254–2018-26).

### Immunosuppression protocol and anti-infective treatment

All recipients benefited from a standard immunosuppression protocol after HTx [13] based on an induction therapy with rabbit antithymocyte globulin (rATG; thymoglobuline; Genzyme, Lyon, France; 1.5 mg.kg^− 1^·day^− 1^ for 5 days) or basiliximab (Simulect, Novartis, Basel, Switzerland) to prevent the onset of an infectious disease. Prophylactic immunosuppressive therapy included calcineurin inhibitors, mycophenolate mofetil and corticosteroids with posology recommended by ISHLT guidelines [14]. In cases of pfDSA, a prophylactic protocol including perioperative management of pfDSA and systematic treatment of subsequent antibody-mediated rejection was applied to patients transplanted with pfDSA. Perioperative management was adapted to the results of the virtual cross-match and to the level of the pfDSA, as evaluated by the mean fluorescence intensity (MFI) of the immunodominant pfDSA (i.e., DSA with the highest MFI) as follows: (1) patients with MFI 500–1000 were treated with IVIg (0.5 g·kg^− 1^ over 4 consecutive days, total dose of 2 g·kg^− 1^; Privigen; CSL Behring AG, Bern, Switzerland), (2) patients with MFI > 1000 were treated with perioperative plasmapheresis sessions (immediately before the HTx, then with 4 sessions over 4 consecutive days using 2/3 fresh frozen plasma and 1/3 albumin, and a fibrinogen substitution was used if the serum fibrinogen < 2 g·L^− 1^) and IVIg. Initiation of the protocol was based on the detection of pfDSA in historical sera. The treatment was readjusted 2 to 3 days after the HTx according to the MFI results at the time of HTx [13]. .Intraoperative antibiotic prophylaxis consisted of the administration of 2 g of cefazolin followed by an additional 1 g every 4 h. Antibiotic prophylaxis was not continued postoperatively. Prophylaxis with valganciclovir and cotrimoxazole was introduced postoperatively from the 6th postoperative day.

### Prevention and diagnosis of pneumonia after HTx

POP was defined as pneumonia occurring between the transplantation and hospital discharge and was the combination of clinical criteria (≥ two criteria including fever > 38.5 °C, hyperleukocytosis > 10^9^·L^− 1^ or leucopoenia < 4·10^8^·L^− 1^, occurrence of purulent secretions and occurrence or persistence of a radiological focus) and microbiological criteria with a positive quantitative culture obtained by bronchial aspiration (threshold ≥10^6^ CFU·mL^− 1^), bronchoalveolar lavage (threshold ≥10^4^ CFU·mL^− 1^) or protected distal sampling (PDP, threshold ≥10^3^ CFU·mL^− 1^) [4, 15]. The diagnosis of herpes virus pneumonia was based on the presence of more than 10,000 copies·mL^− 1^ of the virus and evidence of a cytopathogenic effect on the anatomopathological investigation obtained by the bronchoalveolar lavage. The presence of Candida was considered pathological only if it was associated with lung abscesses. A POP diagnosis was retrospectively confirmed by 2 independent investigators (CV, RP) following analysis of the complete medical file, as previously reported [4]. Recurrent pneumonia was defined by the recurrence of clinical and paraclinical criteria of POP after the improvement of the first POP symptoms with an appropriate antibiotic therapy.

Prevention of POP in the ICU was based on a combination of orotracheal intubation, tracheal balloon pressure maintained between 20 and 30 mmHg, repetitive mouth washing with chlorhexidine every 4 h (Sandoz, Levallois Perret, France) and semirecumbent position. The sedation level was evaluated using the RASS (Richmond Agitation Sedation Scale) score and adapted to obtain a RASS score of 0–1. Patients were extubated as soon as possible even if they received postcardiotomy circulatory support by venoarterial extracorporeal membrane oxygenation (VA-ECMO) for primary graft dysfunction.

### Statistical analyses

Quantitative variables were expressed as the mean (SD) or median (IQR) in non-normally distributed variables. Comparisons between two groups were performed using Student’s t-test or Wilcoxon rank sum test when appropriate. The mean difference between the two groups with the 95% CI was reported for normally distributed variables. Qualitative variables were expressed in numbers (percentages). Comparisons between the two groups were performed using Pearson’s chi-squared test or Fisher’s exact test when appropriate. The absolute risk difference (with 95% CI) was also reported. A Kaplan-Meier survival analysis was used. Log rank tests were then performed on the assumed infection risk factor variables, followed by a univariate Cox regression model including variables with *P* < 0.05.

All *P* values were two-tailed, and *P* < 0.05 was considered significant. Statistical analysis was performed using SAS software (Statistical Analysis System, SAS Institute, Cary, USA) with GraphPad Prism® software (GraphPad Software, Inc., La Jolla, USA) and XLSTAT® software (Addinsoft, Microsoft, Washington, USA).

## Results

In total, 175 patients were transplanted and consecutively included without any patients being lost to follow-up. The recipients were mainly men (73.3%), and the average age was 50 ± 13 years old (Table 1). The indications for HTx were non-ischaemic dilated cardiomyopathy (53%) and coronary artery disease (30%). Overall, 56 patients (32%) were assisted with VA-ECMO until the HTx and were transplanted in salvage conditions, 25 patients (14.2%) were assisted with mechanical circulatory support (MCS) and 15 patients (8.6%) were assisted with mechanical ventilation (Table 1). In the postoperative period, 114 (65.5%) primary graft dysfunctions occurred, requiring VA-ECMO support for 5 [4–8] days. The median mechanical ventilation duration was 2 [1–7] days. The overall survival rate was 92% at 30 days and 87% at 1 year (Table 1).The main causes of death at 30 days and 1 year were septic complications (respectively 64 and 59%) followed by neurological complications (21 and 18%). Six deaths due to infectious complications were attributable to pneumonia.

**Table 1:** Variables associated with postoperative pneumonia (POP) in HTx recipients

Parameters	Cohort	No POP	POP	*P*
N =	175	116	59	
General characteristics				
Recipient age (years)	50 ± 13	53 [41, 60]	54 [47, 62]	0.140
Male recipients (%)	129 (73)	85 (73)	44 (74)	1.000
Prior diabetes mellitus (%)	25	12	13	0.04
Prior cardiac surgery	54 (31)	36 (31)	18 (31)	1
Etiology of heart failure				
-Dilated cardiomyopathy	93 (42)	68 (59)	25 (42)	0.06
-Ischemic cardiomyopathy	53 (30)	31 (27)	22 (37)	0.17
-Other	29 (16)	17 (14)	12 (20)	0.39
Standard allocation (%)	57 (32)	33 (28)	24 (40)	0.12
National high-priority allocation (%)	118 (78)	83 (72)	35 (60)	0.12
*National high-priority allocation 1(%)*	*97 (55)*	*69 (59)*	*28 (47)*	*0.15*
*National high-priority allocation 2 (%)*	*19 (11)*	*13 (7)*	*6 (11)*	*1*
*National high-priority allocation 3 (%)*	*2 (1)*	*1 (1)*	*1 (2)*	*0.44*
Pre-operative data				
VA-ECMO before HTx (%)	56 (32)	37 (32)	19 (32)	1.000
Inotrope (%)	87 (54)	58 (50)	29 (49)	1
vasoconstrictor (%)	10 (6)	5 (4.3)	5 (8.5)	0.31
MV until the HTx (%)	15 (9)	4 (3.5)	11 (19.0)	0.002
VAD or TAH before HTx	25 (14)	18 (16)	7 (12)	0.65
Pre-operative infections (%)	64 (37)	43 (37.1)	21 (35.6)	0.98
Allosensitization (%)	93 (54)	56 (48.3)	37 (62.7)	0.099
Serum creatinine level (mg.dL^−1^)	1.2 ± 0.5	1.14 [0.88, 1.42]	1.16 [0.9, 1.51]	0.469
Prothrombin time (%)	57 ± 18	62.00 [46, 73]	60 [46,69]	0.632
Serum bilirubine level (mg/dL)	2.9 ± 1.9	2.2 [1.4, 3.8]	2.7 [1.4, 3.6]	0.705
Per operative data				
Cold ischemia time (min)	191 ± 53	210 [168, 233]	192[156, 216]	0.080
ECC duration (min)	121 ± 39	110 [95,135]	110 [98, 142]	0.918
Intraoperative transfusion (%)	133 (76)	82 (74.5)	51 (87.9)	0.067
Post-operative data				
VA-ECMO post-HTx (%)	114 (66)	68 (58.6)	47 (79.7)	0.009
Revision surgery (%)	39 (22)	20 (17.5)	19 (33.3)	0.033
SAP 2 score in ICU	43 (±15)	40 [30,50]	50 [34,58]	0.02
SOFA score in ICU	6 (±3)	6[4,8]	6 [4,8]	0.70
Post-HTx transfusion (%)	60 (34)	25 (21.7)	35 (59.3)	< 0.001
Immunosuppressive therapy				
­Plasmapheresis (%)	69 (39)	40 (34.5)	29 (49.2)	0.087
­Polyvalent Immunoglobulin(%)	85 (48)	54 (46.6)	31 (53.4)	0.486
­Anti-thymocyte globulin (%)	169 (95)	111 (95)	56 (94.9)	1.000
­Basiliximab (%)	6 (5)	5 (4.3)	1 (1.7)	0.665

Qualitative data are expressed in percentage and qualitative data are expressed on average with standard derivation for the global cohort and on median with 1st and 3th quartile for recipients with and without post-operative pneumonia. *POP* Post-operative pneumonia, *VA ECMO* Veno -arterial Extracorporeal Membrane Oxygenation, *HTx* Heart transplantation, *MV* Mechanical ventilation, *VAD* Ventricular assisted devices, *TAH* Total artificial heart, *ECC* Extracorporeal circulation, *SAP 2 score* Simplified Acute Physiology 2 score, *SOFA score* Sequential Organ Failure Assessment score, *ICU* Intensive care unit

Prior to hospital discharge, 89 instances of POP occurred in 59 (33.7%) HTx recipients (Table 2), of whom 19 recipients developed recurrent POP (two or more episodes of POP). The mean time between HTx and POP was 4 (IQR: 3–6) days. The main pathogens involved were *Enterobacteriaceae* (53%), mainly *Klebsiella pneumonia* (21%) and *Pseudomonas aeruginosa* (36%). In 25% of cases, POP was polymicrobial (Table 2). Bacterial pathogens that are classically associated with early POP such as *Streptococcus pneumoniae*, *Haemophilus influenza* and *Staphylococcus aureus* were responsible for a small proportion (10%) of POP in this cohort, and cefotaxime was effective in only 36% of the cases (36% of *Pseudomonas aeruginosa* and 69% of *Enterobacteriaceae* produced extended-spectrum or AmpC beta-lactamases). In the specific cases of the 93 sensitized recipients (53%), 69 patients (74%) were transplanted despite a pfDSA higher than 1000 MFI (Table 1). Thirty-seven sensitized recipients developed POP compared to the 22 other recipients (40% vs 27%, respectively, *P* = 0.08).

**Table 2:** Pathogens associated with postoperative pneumonia in HTx recipients

Pathogens	N (%)
Oropharyngeal flora	16 (18)
*Haemophilus influenza*	5 (6)
*Streptococcus pneumonie*	1 (1)
*Staphylococcus aureus*	1 (1)
*Enterococcus faecalis*	1 (1)
*Enterococcus faecium*	1 (1)
*Enterobacteriaceae*	48 (53)
*Escherichia coli*	2 (2)
*Proteus mirabilis*	2 (2)
*Klebsiella pneumoniae*	19 (21)
*Klebsiella oxytoca*	1 (1)
*Citrobacter koseri*	1 (1)
*Enterobacter aerogenes*	5 (6)
*Enterobacter cloacae*	8 (9)
*Citrobacter freundi*	5 (4)
*Morganella morganii*	1 (1)
*Serratia marcescens*	3 (3)
*Hafnia alvei*	1 (1)
Non-fermentative Gram negative bacilli	34 (38)
*Stenotrophomonas maltophilia*	2 (2)
*Pseudomonas aeruginosa*	32 (36)
*Acinetobacter baumanii*	0 (0)
Nonbacterial pathogens	4 (4)
*Herpes Simplex Virus*	1 (1)
*Candida albicans*	1 (1)
*Aspergillus fumigatus*	2 (2)
Non-documented infections	2 (2)

Data expressed on number and percentage of post-operative pneumonia

The multivariate analysis showed that mechanical ventilation at the time of HTx (OR: 1.42, 95% CI [1.12–1.80], *P* < 0.01) and postoperative blood transfusion (OR: 1.42, 95% CI [1.20–1.70], *P* < 0.01) were the main risk factors for POP. Plasma exchanges and IVIg were not associated with an increased risk of POP.

**Fig. 1:**
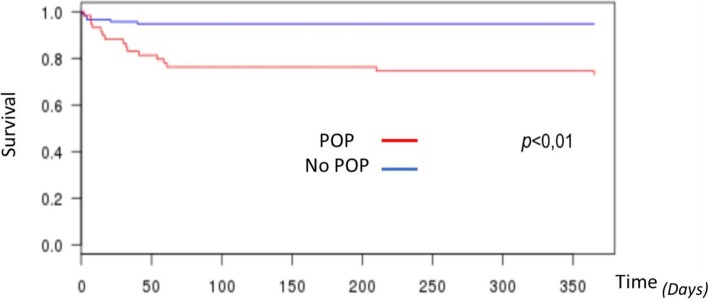
One-year survival curve of HTx recipients with and without postoperative pneumonia (POP), respectively 95% versus 73%, *p < 0.01*

In the group of HTx patients with POP, mechanical ventilation duration, postoperative VA-ECMO support and length of stay in the hospital ICU were increased in comparison with those in the group of HTx patients without POP. In the patients with POP, mortality was increased significantly at 30 days (OR: 4 [1.3–12.4], *P* = 0.01) and at 1 year (OR: 6.8 [2.5–18.4], *P* < 0.01) (Fig. 1). Nineteen recipients developed recurrent POP. Their 30-day and 1-year mortalities were similar to the recipients with only 1 episode of POP (respectively OR: 1.1 [0.2–7.6], *P* = 1 and OR. 0.8 [0.2–3], *P* = 0.75). In contrast, POP does not increase the rejection rate at 1 year (Table 3).

**Table 3:** Comparison of secondary outcomes between HTx recipients with and without postoperative pneumonia

Parameters	No POP (*n* = 116)	POP (*n* = 59)	OR (IC_95%_)	*p*
Duration of mechanical ventilation (days)	1 [1,3]	9 [4,23]	–	< 0,01
VA-ECMO Duration (days)	4 [1,10]	10 [5,16]	–	0,01
ICU length of stay (days)	16 [11,23]	26 [15,41]	–	< 0,01
Hospital length of stay (days) –	29 [20,40]	45 [28,63]	–	< 0,01
Kidney replacement therapy, n (%)	20 (17)	38 (66)	9,0 (4,3 – 16,6)	< 0,01
One-year rejection, n (%)	58 (50)	30 (50)	1.01 (0,5 – 1,9)	1,00
30-day mortality, n (%)	5 (4)	9 (15)	4,0 (1,3 – 12,4)	0,02
One-year mortality, n (%)	6 (5)	16 (27)	6,8 (2,5–18,4)	< 0,01

Qualitative data are expressed in percentage and qualitative data are expressed on median with 1st and 3th quartile for recipients with or without post-operative pneumonia (POP)

## Discussion

In this cohort of HTx recipients, a POP occurred in 33.7% of patients and was mainly caused by *Enterobacteriaceae* or *Pseudomonas aeruginosa*, and consistently increased mortality at 30 days and 1 year. Preoperative mechanical ventilation and postoperative blood transfusion, which were indirectly linked to postoperative bleeding, were identified as the main risk factors for POP after the HTx.

In a Californian cohort of 620 HTx recipients, infections were the main cause of morbidity and mortality, while bacterial pathogenesis was involved in only 43% of cases [8]. In addition, most of the instances of pneumonia occurring later after the HTx were due to *Cytomegalovirus*, *Aspergillus fumigatus* and *Pneumocystis carinii* in another study [7]. However, although both of these reports concern infectious events in immunocompromised patients, there are not focused solely on the early postoperative period. In our cohort, almost all of the POP cases were bacterial. Community germs (Oropharyngeal flora, *Streptococcus pneumoniae*, *Haemophilus influenza* and *Staphylococcus aureus*) were responsible for only a small proportion of pneumonia (10%), and the most frequently implicated organisms in POP were *Enterobacteriaceae* (53%) or *Pseudomonas aeruginosa* (36%). Such a microbiology of POP pathogens is similar to the one observed following other cardiac surgeries [6]. .This point should be considered when choosing the appropriate empirical antimicrobial therapy for pneumonia in HTx recipients within the perioperative period. Infections with “opportunistic” pathogens were rare (3 pulmonary aspergillosis, 1 pulmonary abscess with *C. albicans* and 3 *HSV* pneumonias) and appeared late in the infectious history and mainly in the patients with recurrent pneumonia. Therefore, the administration of unusual anti-infective drugs, such as antifungals or antivirals, must be based on clinical and paraclinical arguments and not solely on the induced immunosuppression.

A large predominance of pathogens from the digestive tract (*Enterobacteriaceae* and *Pseudomonas aeruginosa)* can be explained by the bacterial translocation phenomena following cardiac surgery [16, 17]. .Indeed, haemodynamic instability, low cardiac output and systemic inflammatory response syndrome induced during cardiac surgery can lead to intestinal ischaemia and bacterial translocation [16]. Moreover, recipients with POP are assisted more by VA-ECMO in the postoperative period due to haemodynamic instability or low blood flow during the transplantation that may predispose patients to infections [10].

More than two-thirds of our recipients were assisted by VA-ECMO after transplantation, and although patients with postoperative pneumonia appeared to be more assisted, VA-ECMO does not appear to be a risk factor for POP. Nosocomial infections, especially ventilator-associated pneumonia, are frequent in patients assisted by VA-ECMO [18], and HTx recipients assisted by VA-ECMO are known to develop nosocomial infections and pneumonia [10]. Moreover, the low cardiac output syndrome may explain the increased incidence of POP after HTx in comparison with conventional cardiac surgery (33.7% vs 5.7%), in which the incidence of intra- and postoperative haemodynamic instability is less important [4]. In our cohort, a large proportion of recipients presented risk factors for primary graft dysfunction, such as allosensitization (54%), preoperative VA-ECMO support (32%) or mechanical ventilation at the time of transplantation (9%) [19–21].

On the contrary, preoperative VA-ECMO is an efficient temporary device, and its early use is efficient for preventing organ failure, especially in the occurrence of renal insufficiency. In our cohort, 68% of the recipients benefited from an emergency graft attribution, and 32% were assisted by VA-ECMO before transplantation. Based on the institutional protocol, all patients with VA-ECMO (whether or not in a transplant project) are extubated as soon as possible to prevent the risk of pneumonia and ICU weakness. Early extubation allows neurological evaluation and participation in physiotherapy to select the best candidates for transplant projects. Thus, unlike the international registries, the use of VA-ECMO before transplantation did not appear to be a risk factor for POP in our study [3]. Altogether, these findings suggest that tissue hypoperfusion occurring during low cardiac output syndrome could contribute to digestive bacterial translocation and may influence the pathogens involved in early POP in HTx recipients.

The association of increased incidence of POP and transfusion or reoperation for bleeding are additional arguments to explain the blood-forming origin of POP [10] and this unusual high rate of *Enterobacteriaceae* and *Pseudomonas aeruginosa* in POP after HTx. Transfusion per se is an additional process that is known to increase the rate of postoperative infections after the first transfusion of packed red blood cells [22] and to increase 28-day mortality [23]. The modulation of the immune response by transfusion may promote the development of postoperative infectious complications [24, 25]. A transfusion in patients supported with VA-ECMO is also known to increase infectious complications [26]. In the present study, beyond the risk associated with low cardiac output and surgical reoperation itself, 34% of HTx recipients received a postoperative transfusion despite a restrictive strategy of blood transfusion in our institution, thereby increasing the risk of postoperative pneumonia. Anticipation of perioperative bleeding disorders and meticulous surgical haemostasis are necessary to prevent haemorrhagic risks.

In this work, preoperative mechanical ventilation also appears to be a major risk factor for POP after HTx and is already known to be a risk factor for healthcare-related pneumonia and mortality, regardless of the context of HTx [3, 27]. The bacterial colonization of the tracheobronchial tree and alteration of fluid clearance are well known and have been reported to be the main pathophysiological mechanisms of pneumonia during mechanical ventilation [28]. Regarding the effect of mechanical ventilation on POP in HTx recipients, a haematogenic mechanism is more likely to be involved. Therefore, mechanical ventilation as a major risk factor might be reflective of the HTX recipients’ postoperative severity, given that fast-track management is the priority in all HTx recipients, including those with VA-ECMO support after the transplantation.

Plasmapheresis has been shown to prevent AMR in sensitized recipients [11, 12]. However, this more aggressive immunosuppression therapy may increase the recipients’ exposure to infectious complications [29]. In kidney transplantation, Chung et al. showed that the administration of rituximab and plasma exchanges increased the risk of postoperative infectious complications [29]. In the present study, HTx recipients treated with plasmapheresis and/or IVIg treatment did not have more instances of POP than other HTx recipients, suggesting that this therapy is not the main mechanism involved in sensitized HTx recipients.

Our work has several limitations. First, this is a retrospective and monocentric study with a relatively small sample size. Nevertheless, none of the patients were lost to follow-up, and no data are missing from this cohort. Second, the population studied is relatively unusual, with a high rate of sensitized recipients receiving HTx. However, the proportion of sensitized recipients is increasing, possibly because the technological means to detect pfDSA are evolving. Third, the proportion of HTx recipients assisted with postoperative VA-ECMO support is coherent in this study. Due to the rarity of heart grafts and to the graft distribution prioritization programme, the number of primary graft dysfunctions as well as the preoperative severity of recipients is increasing. Nevertheless, our study provides important information on this type of population.

## Conclusion

The occurrence of pneumonia after HTx is frequent and increases mortality among HTx recipients. Most frequently, the pathogens are *Enterobacteriaceae* or *Pseudomonas aeruginosa*, which should be at the centre of the empirical treatment for POP. In contrast to plasmapheresis and IVIg administration, mechanical ventilation prior to HTx and postoperative transfusion appear to be the main identified risk factors for POP.

## Supplementary information

**Additional file 1.** Newletter for patients

## Data Availability

The datasets used and/or analysed during the current study are available from the corresponding author on reasonable request. Drs Amour and Vidal had full access to all of the data in the study and take responsibility for the integrity of the data and the accuracy of the data analysis.

## Abbreviations

CI: Confidence Interval
HTx: Heart Transplantation
ICU: Intensive Care Unit
IQR: Inter Quartile Range
IVIg: IntraVenous Immunoglobulins
MFI: Mean Fluorescence Intensity
OR: Odd Ratio
pfDSA: Preformed Donor-Specific anti-HLA Antibodies
POP: Post Operative Pneumonia
RASS: Richmond Agitation-sedation Scale
rATG: Rabbit AntiThymocyte Globulin
SD: Standard Deviation
VA-ECMO: Veno-Arterial Extracorporeal Membrane Oxygenation
