# Supplementary material for: Predictive risk factors for postoperative pneumonia after heart transplantation
Source: BMC Anesthesiol. 2020 Jan 7;20:8. doi: 10.1186/s12871-019-0923-3 (PMC6947950; doi:10.1186/s12871-019-0923-3)
Supplement: Supplementary file 1 — Additional file 1. Newletter for patients [file 12871_2019_923_MOESM1_ESM.pdf]

NOTE D'INFORMATION
DESTINE AU PATIENT

Lisez attentivement ce document.

Vous pourrez alors décider si vous souhaitez ou non vous opposer à l'utilisation de vos prélèvements existants et de vos données de santé disponibles dans votre dossier médical.

<u>TITRE DU PROJET</u> : Facteurs de risque des pneumonies post opératoires après transplantation cardiaque.

<u>Nom et adresse du Promoteur</u>: Groupe Hospitalier Pitié Salpêtrière, 47 – 83 boulevard de l'Hopital, 75013 Paris

<u>Centres de la Recherche</u> :
Groupe Hospitalier Pitié Salpêtrière, 47 – 83 boulevard de l'Hopital, 75013 Paris
Centre Hospitalier Régional Felix Guyon, Réanimation polyvalente, Allée des Topazes, 97400, La Réunion

Madame, Monsieur,

Dans le but d'améliorer les connaissances sur les complications infectieuses et les pneumonies survenant dans les suites d'une transplantation cardiaque, l'Institut de Cardiologie du GH Pitié Salpétrière a mis en place un projet de recherche clinique décrivant rétrospectivement l'épidémiologie les infections en post opératoire de transplantation cardiaque

Vous avez fait, par le passé, l'objet d'une hospitalisation pour ce motif.

C'est pourquoi, le Dr VIDAL Charles, souhaiterait utiliser vos prélèvements et les données de santé disponibles dans votre dossier médical en lien avec cette surinfection.

Votre participation à cette recherche est volontaire.
Si vous ne souhaitez pas participer, vous n'avez pas à vous justifier, et cela n'aura aucune conséquence sur votre prise en charge ou sur la relation avec le médecin.

<u>Quel est l'objectif de la recherche</u> ?

Cette étude rétrospective concerne des patients adultes ayant bénéficié d'une transplantation cardiaque. Elle a pour objectif de recueillir des données épidémiologiques sur ce type de pathologie.

Quelles sont les données nécessaires à cette recherche ?

Les besoins de la recherche nécessitent le recueil des données cliniques et paracliniques en rapport avec votre hospitalisation lors de la transplantation cardiaque. Les données recueillies seront dé-identifiées et seront codées de manière à préserver votre anonymat.

Comment seront traitées mes données ?

Dans le cadre de cette recherche non interventionnelle, dans laquelle le GH Pitié Salpétrière souhaite utiliser vos données de santé, un traitement de vos données personnelles va être mis en œuvre pour permettre d'analyser les résultats de la recherche au regard de l'objectif de cette dernière qui vous a été présenté, dans les conditions garantissant leur confidentialité.

A cette fin, les données médicales vous concernant comprenant les données relatives à votre hospitalisation ainsi qu'à vos antécédents médicaux, seront recueillies à partir de votre dossier médical et entièrement dés-identifiées (sans votre nom-prénom) puis enregistrées dans une base de données informatisée avec un numéro de code. Toutes les données recueillies à l'occasion de la présente recherche seront analysées

Toutes les données vous concernant et utiles à l'étude disponibles dans votre dossier médical, pourront être consultées par les personnes soumises au secret professionnel qui collaborent à cette recherche.

Quels sont mes droits ?

Votre participation à cette étude est volontaire. Vous êtes entièrement libre de participer ou non à cette étude.

Conformément aux dispositions de loi relative à « l'informatique, aux fichiers et aux libertés » du 06 janvier 1978 modifiée, vous disposez d'un droit d'accès et de rectification. Vous disposez également d'un droit d'opposition à la transmission des données couvertes par le secret professionnel susceptibles d'être utilisées dans le cadre de cette recherche et d'être traitées. Ce droit peut être appliqué sans justification de votre part et est sans conséquence
sur votre prise en charge ou votre relation avec le médecin.

Les résultats globaux de la recherche pourront vous être transmis par le Dr VIDAL Charles si vous lui en faites la demande.

Vous pouvez exercer ces droits en adressant un courrier au Dr VIDAL Charles dont les coordonnées sont précisées ci-dessous :

Dr VIDAL Charles
Centre Hospitalier Régional Felix Guyon
Réanimation polyvalente
Allée des Topazes, 97400, La Réunion

Vous pouvez également accéder directement ou par l'intermédiaire d'un médecin de votre choix à l'ensemble de vos données médicales en application des dispositions de l'article L 1111-7 du Code de la Santé Publique.

Sans retour de votre part dans un délai d'un mois à compter de la date de réception de cette note d'information, les données de santé disponibles dans votre dossier médical et vos prélèvements seront utilisées pour cette recherche.

Si vous avez des questions ou souhaitez d'autres informations, vous pouvez contacter le :

Dr VIDAL Charles
Centre Hospitalier Régional Felix Guyon
Réanimation polyvalente
Allée des Topazes, 97400, La Réunion
charlesvidal@orange.fr
